# Malvidin induces hepatic stellate cell apoptosis via the endoplasmic reticulum stress pathway and mitochondrial pathway

**DOI:** 10.1002/fsn3.1810

**Published:** 2020-08-10

**Authors:** Yanhong Ma, Yahui Li, Hongzhi Zhang, Ying Wang, Caie Wu, Wuyang Huang

**Affiliations:** ^1^ Institute of Agro‐Product Processing Jiangsu Academy of Agricultural Sciences Nanjing China; ^2^ School of Food and Biological Engineering Jiangsu University Zhenjiang China; ^3^ College of Light Industry and Food Engineering Nanjing Forestry University Nanjing China; ^4^ Jiangsu Key Laboratory for Horticultural Crop Genetic Improvement Jiangsu Academy of Agricultural Sciences Nanjing China

**Keywords:** apoptosis, endoplasmic reticulum stress, hepatic stellate cells, malvidin, mitochondrial pathways

## Abstract

Blueberries have great beneficial effects due to high level of anthocyanins, especially malvidin. Hepatic stellate cells (HSCs) can be activated and increase excessive extracellular matrix (ECM) components, which play a central role in liver fibrogenesis. Therefore, activated HSC’s apoptosis can be induced to recover liver fibrosis. Malvidin's effects on apoptosis in rat activated hepatic stellate T6 cells (HSC‐T6) in vitro were investigated here. High concentration of malvidin was found to significantly induce apoptosis, activate caspase‐3, increase malondialdehyde, upregulate Bax, but downregulate Bcl‐2. Moreover, malvidin upregulated the protein levels of some endoplasmic reticulum stress (ERS)‐typical markers, including caspase‐12, glucose‐regulated protein 78 (GRP78), and CCAAT/enhancer‐binding protein (C/EBP) homologous protein (CHOP), suggesting that malvidin induced HSC apoptosis by the ERS apoptosis pathway as well as the mitochondrial‐dependent pathway. These findings indicated that blueberry anthocyanins, especially malvidin, could induce activated hepatic stellate cell apoptosis and might act as one kind of functional food ingredient or a novel nutraceutical beneficial for liver health.

## Graphical abstract

Malvidin increased reactive oxygen species and malondialdehyde levels in hepatic stellate T6 cells. Malvidin downregulated Bcl‐2, upregulated Bax, and activated caspase‐3. Malvidin induced HSC apoptosis by mitochondrial and endoplasmic reticulum stress pathway.

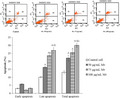

## 1 INTRODUCTION

Liver fibrosis, the accumulation of excessive extracellular matrix (ECM) in the liver parenchyma, is one main predictor for most chronic liver diseases (Ni et al., 2018). Hepatic stellate cells (HSCs) have a crucial role in liver fibrosis development (Puche, Saiman, & Friedman, 2013). In normal liver, HSCs stay in a quiescent state, whose main function is retinoid storage (Friedman, 2008). Stimulations, such as growth factors and reactive oxygen species (ROS), can activate HSCs during hepatic fibrogenesis. Activated HSCs change phenotype into myofibroblast‐like cells, lose retinoid droplets, and increase contractility, proliferation, and ECM synthesis (Muriel, 2009; Rippe & Brenner, 2004). Therefore, activated HSCs are recognized as one major source of collagenous and/or non‐collagenous matrix proteins accumulated in the fibrotic liver (Gaҫa et al., 2003). Thus, the inhibiting HSC’s activation or inducing activated HSCs’ apoptosis provides a novel strategy for anti‐fibrotic treatment (Higashi, Friedman, & Hoshida, 2017).

Anthocyanins are one type of flavonoids that widely distributed in many dietary plants, like fruits, beans, cereals, and vegetables, especially blueberries. Blueberries possess higher anthocyanin contents and stronger antioxidant activity than most other fruits and vegetables (Hosseinian & Beta, 2007; Reque et al., 2014). Many reports have confirmed that anthocyanins exhibit powerful bio‐activities, including antioxidant, anti‐inflammatory, anti‐tumor, and hepatoprotective properties in vitro or in animal models (Mazewski, Liang, & de Mejia, 2017; Sun et al., 2018). So anthocyanins could be considered as one of the best physiologically functional phytochemicals (Prior et al., 1998).

The health benefits of anthocyanins were associated with the anthocyanin quantity via dietary intake. However, so far there is still no definite recommended daily intake of anthocyanins for optimal health in China or other countries. Recent researches have pointed out the average daily intake of anthocyanins ranged from 19.8 to 82 mg/day in different countries, involving China, Australia, and Europe (Li et al., 2013; Murphy, Walker, Dyer, & Bryan, 2019; Zamora‐Ros et al., 2011). For men, the average daily anthocyanin intake has been 19.83 mg/day in Holland and 64.88 mg/day in Italy, while for women ranged from 18.73 mg/day in Spain to 44.08 mg/day in Italy. In Finland, the consumption has already been estimated to be 82 mg/day (Morais, de Rosso, Estadella, & Pisani, 2016). The continuous research on anthocyanins will undoubtedly provide a basis for pursing healthy dietary guidance recommendations.

The malvidin, one of the primary plant pigments mainly existing in fruit skins, has been proved the most abundant anthocyanin of blueberries, in 3‐position glycosylated forms, for example, malvidin‐3‐galactoside and malvidin‐3‐glucoside (Hosseinian & Beta, 2007). Like other anthocyanins, malvidin‐dependent biological effects may be of great significance. Malvidins have antioxidant, anti‐hypertensive, anti‐inflammatory, anti‐obesity, anti‐osteoarthritis, and anti‐proliferative effects (Baba, Nivetha, Chattopadhyay, & Nagini, 2017; Huang, Wang, Liu, Zheng, & Li, 2014; Saulite, Jekabsons, Klavins, Muceniece, & Riekstina, 2019). In addition, malvidins exhibit cytotoxicity against some cancer cells; therefore, they also have potential to be as anticancer drugs (Mazewski, Liang, & de Mejia, 2018; Yoshino et al., 2018). Hwang, Choi, Choi, Chung, and Jeong (2011) reported that the anthocyanin extracts from purple sweet potatoes had a potent hepatoprotective effect in the mouse liver damage model induced by tert‐butyl hydroperoxide. However, the malvidin's effects on HSCs and hepatic fibrosis have still been explored poorly. Our preliminary studies revealed that malvidins selectively inhibited cell proliferation in HSCs, but not in normal live cells (unpublished data). This may provide a basis for the anti‐hepatic fibrosis effects of blueberry malvidins. In this study, the malvidin's effects on rat activated HSC‐T6 cell apoptosis were investigated, while its possible mechanism was revealed.

## 2 MATERIALS AND METHODS

### 2.1 Chemicals and reagents

Malvidin (Mv) and 3‐(4,5‐dimethylthiazol‐2yl‐)‐2,5‐diphenyl tetrazolium bromide (MTT) were bought from Sigma. Fetal bovine serum (FBS) and Dulbecco's modified Eagle's medium (DMEM) were bought from GIBCO. Bicinchoninic acid (BCA), Malondialdehyde (MDA), Superoxide dismutase (SOD), reduced glutathione (GSH)/oxidized glutathione disulfide (GSSG), and lactate dehydrogenase (LDH) assay kits were bought from Jiancheng Bioengineering Institute (Nanjing, China). Hoechst 33258 dye and 2′,7′‐dichlorofluorescin diacetate (DCFH‐DA) detection kit were bought from Beyotime Institute of Biotechnology (Haimeng, China). Annexin V/PI apoptosis kit was bought from MultiSciences Biotech Co., Ltd. The Electro‐Chemi‐Luminescence (ECL) chemiluminescence reagent was bought from Pierce Biotechnology Inc. All the reagents or chemicals were in analytical grade.

### 2.2 Antibodies

The primary antibodies, including rabbit anti‐β‐actin (sc‐47778), rabbit anti‐bax (sc‐493), rabbit anti‐Bcl‐2 (sc‐492), rabbit anti‐cleaved caspase‐3 (sc‐7148), rabbit anti‐pro‐caspase‐12 (sc‐5627), rabbit anti‐CHOP/GADD153 (sc‐793), and rabbit anti‐GRP78 (sc‐13968), were obtained from Santa Cruz Biotechnology Inc. Secondary antibodies, goat anti‐mouse/anti‐rabbit IgG‐horseradish peroxidase (HRP; BA1054) conjugated antibodies, were bought from Boster Biotechnology Inc. All the primary antibodies were diluted 1,000 folds except that anti‐Bax was 1:500 and anti‐GRP78 was 1:200, while the secondary antibodies were all diluted 2,000 folds.

### 2.3 HSC‐T6 cell culture and treatment

The activated HSC‐T6 rat stellate cell lines were bought from Shanghai Institute of Biochemistry and Cell Biology, Chinese Academy of Sciences (Shanghai, China). HSC‐T6 cells were incubated in DMEM containing 1% penicillin‐streptomycin and 10% FBS until the mid‐log phase. The cells were cultured in a 5% CO_2_ humidified atmosphere at 37°C. The cells in 6‐well plates were stimulated with Mv in DMEM at the final concentrations of 25, 50, 75, 100, 125, or 150 μg/ml for 24, 48, and 72 hr, respectively. The cell model treated with different Mv concentrations was established according to our preliminary experiment (Y. H. Ma, & W. Y. Huang, unpublished data) and other reports. The control was DMEM. ELISA analysis for the supernatants and Western bolt for the cells were conducted.

### 2.4 Cell viability assay

HSC‐T6 cell's viability was measured by the MTT method (Zhu et al., 2012). The cells in 96‐well microplate (2 × 10^4^ cells/ml) were incubated with or without different Mv concentrations for 24, 48, and 72 hr. Then each well added a 20 μl of 5 mg/ml MTT to incubate for 4 hr. A 150 μl of DMSO was added after removing the supernatant, and the well was gently shaken for 10 min at 37°C. The absorbance at 490 nm was detected using a StatFax‐2100 Microplate Reader (Awareness Technology Inc.). DMEM was a blank. The following formula was used to calculate: cell viability = (test OD_490_ − blank OD_490_)/(control OD_490_ − blank OD_490_) × 100%.

### 2.5 ELISA assay

Lactate dehydrogenase assay was conducted by the LDH enzyme activity kit. The marker of lipid peroxidation and MDA concentration were measured according to the MDA assay kit manual. Superoxide dismutase and GSH/GSSG levels were also detected using the corresponding test kits. The absorbance at 450 nm was detected on a Synergy H4 Multi‐Mode Microplate Reader (BioTek Instruments, Inc.) using Hyper Terminal Applet ELISA software.

### 2.6 Fluorescence microscopy (FM) and transmission electron microscopy (TEM)

HSC‐T6 cells were stained with 10 μmol/L Hoechst 33258 dye for 15 min. The stained cells were imaged using an IX53 Inverted Fluorescent Microscope (Olympus) with 460 nm emission and 350 nm excitation filters. The nuclear DNA was blue fluorescent. The number of cells with apoptotic nuclear condensation was counted in each well. The TEM was performed following the method previously reported (Lee & Friedman, 2011) by a FEI Tecnai G2 Spirit Bio TWIN Microscopes Electron Microscopy (FEI).

### 2.7 Flow cytometric assay on apoptosis

HSC‐T6 cells were in the incubation with 0, 50, 75, or 100 μg/ml of malvidin for 24 hr. The collected cells were stained by 20 μl of AnnexinV‐fluorescein isothiocyanate (FITC), 20 μl of propidium iodide (PI), and 100 μl of binding buffer (Roche), which were incubated in the dark for 15 min at room temperature (Zhu et al., 2014). Samples were immediately analyzed on a Becton Dickinson (BD) FACS Calibur Flow Cytometer (BD Biosciences) at 488 and 546 nm. The early apoptotic cells were those with visible AnnexinV (green) but without PI (red) staining, while the late apoptotic cells were those only with PI staining, respectively. The percentage of apoptosis was calculated.

### 2.8 Reactive oxygen species (ROS) assay

The HSC‐T6 cell's ROS levels were measured by DCFH‐DA detection kit (Beyotime). Briefly, after Mv treatment (final concentrations: 0, 50, 75, and 100 μg/ml, respectively) for 24 hr, HSC‐T6 cells were stained with 10 μM/ml of H_2_DCFDA at 37°C for 20 min in the dark. The relative ROS levels in the HSC‐T6 cells were observed by an IX53 Inverted Fluorescent Microscope (Olympus). Fluorescent intensity was measured at the 530 nm emission wavelength and 485 nm excitation wavelength, respectively.

### 2.9 Western blot

The total protein in HSC‐T6 cells was detected by a BCA protein assay kit. Each 10 μg of protein was separated using 10% SDS**‐**PAGE, and then transferred to a polyvinylidene difluoride (PVDF) membrane (BIO‐RAD). The primary antibodies and secondary antibodies were added orderly after blocking with 5% non‐fat dry milk. The protein bands were determined by Pierce ECL substrate kit, and the levels of proteins were obtained using a LAS‐3000 imaging system (Fuji) controlled by a Gel‐Pro analyzer software. The untreated cell's lysate was loaded on each gel as a control. Data were expressed as the fold over the control normalized by β‐actin.

### 2.10 Statistical analysis

All the results were calculated as mean value of triplicate experiments ± standard deviation (*SD*). The figures were from Microsoft Excel 2003. Comparison between two groups was performed with *t* test, and comparison among multiple groups was done using analysis of variance (ANOVA). Statistical significance was acceptable when *p* < .05.

## 3 RESULTS

### 3.1 Malvidin inhibited HSC‐T6 cell proliferation

**Figure 1:**
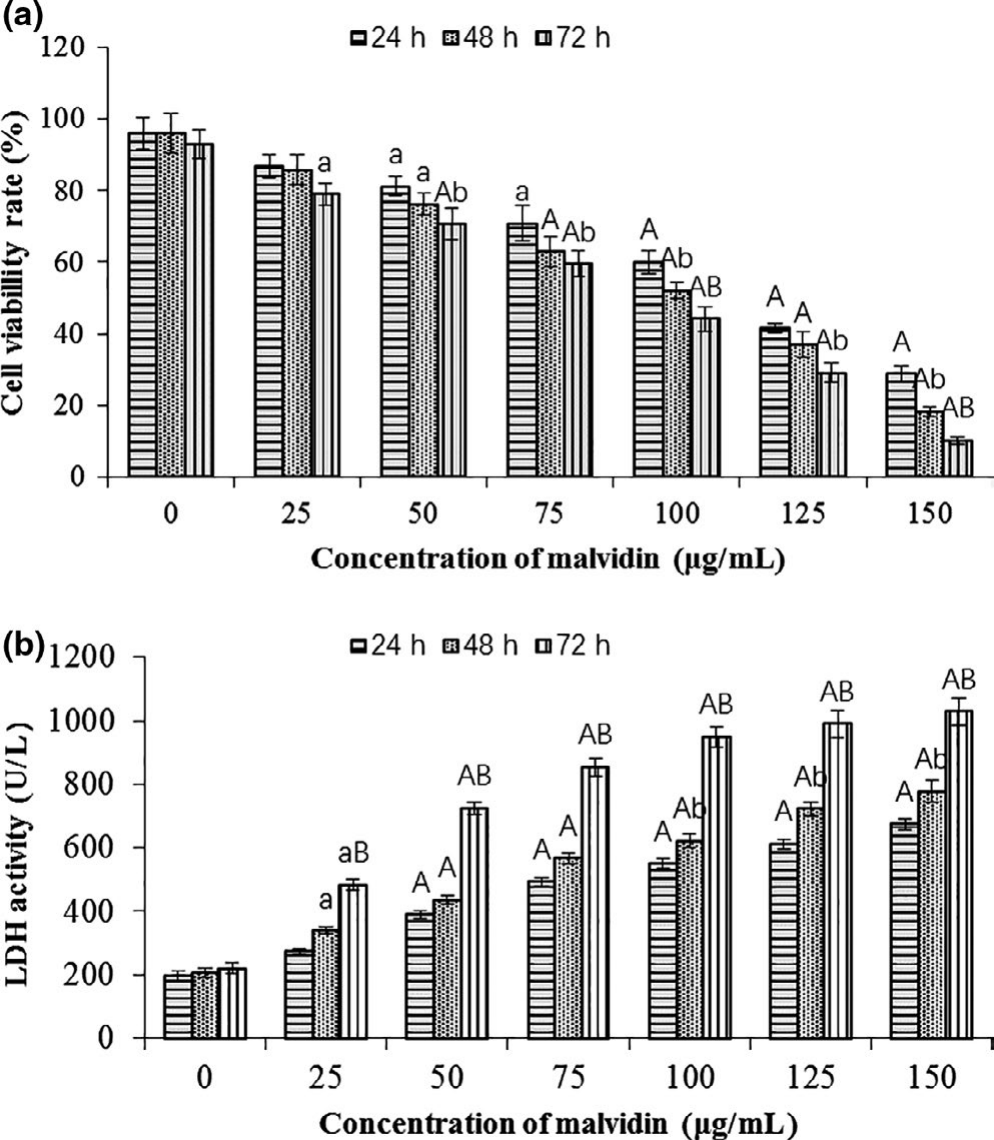
Effects of malvidin on HSC‐T6 cell growth. HSC‐T6 cells were incubated with different concentrations of malvidin for 24, 48, or 72 hr. The cell proliferation capability was examined by MTT assay (a). The necrosis of HSC cells was measured using the LDH activity assay (b). Data are presented as mean ± *SD*. ^a^ *p* < .05, ^A^ *p* < .01 compared to the control at the same concentration; ^b^ *p* < .05, ^B^ *p* < .01 compared group of 24 hr with the same concentration

Different concentrations of malvidin's effects on the HSC‐T6 cell proliferation were evaluated by a MTT assay. Malvidins inhibited the viability of activated HSC‐T6 cells in a dose‐dependent and time‐dependent manner (Figure 1a). Malvidin at concentration of 50 μg/ml could significantly decrease the cell proliferation with HSC cell viability rates down to 81.24 ± 2.66% at 24 hr and 76.22 ± 3.10% at 48 hr (both *p* < .05), and 70.70 ± 4.33% at 72 hr (*p* < .01), respectively. As the concentration above 50 μg/ml, malvidins showed more pronounced inhibition (*p* < .01), and a long‐time stimulation (72 hr) had a significantly stronger inhibitory effect (*p* < .05) than a short‐time stimulation (24 hr). The cell viability rate was only 10.21 ± 1.06% for the treatment with 150 μg/ml malvidin for 72 hr.

Lactate dehydrogenase, one kind of stable cytosolic enzymes in necrotic cells, is released after membrane damaging, which is an important indicator to assess cell necrosis and could only be detected in necrotic cells (Schueren et al., 2014). Here, LDH analysis was used to determine the HSC‐T6 cell necrosis and showed similar results with the MTT assay (Figure 1b). It exhibited extremely significant difference to the control at the concentration more than 50 μg/ml (*p* < .01). Malvidin of all the concentrations had a significantly higher LDH activity at 72 hr than that at 24 hr (*p* < .01). The LDH activity of HSC‐T6 cells treated with 150 μg/ml malvidin was about 3–5 folds of the control. These data suggested that malvidins induced the necrosis of HSC cells, which coincided well with the result of the MTT assay.

### 3.2 Malvidin destroyed HSC‐T6 cell morphology

**Figure 2:**
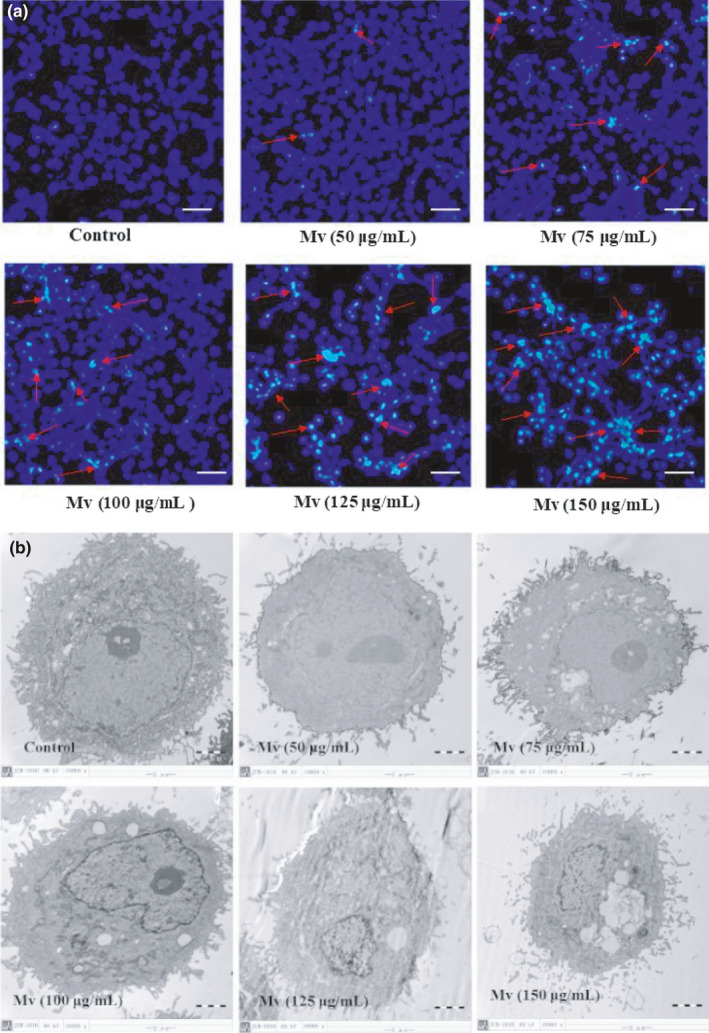
Morphological changes of HSC‐T6 cells including fluorescence microscopy (FM) observation (a) and transmission electron microscopy (TEM) observation (b) were conducted after the incubation with different concentrations of malvidin for 24 hr. A representative set of images from three independent experiments is shown. The nuclear DNA shows blue fluorescence. The red arrows indicate apoptosis HSC‐T6 cells showing nuclear condensation. All images presented are in 200× magnification for FM, and in 10,000× magnification for TEM. Each scale bar is 50 µm

Fluorescence microscopy observation and TEM observation showed morphological changes in HSC‐T6 cells with the stimulation by different concentration malvidin for 24 hr. The HSC‐T6 cells with malvidin above 75 μg/ml exhibited early apoptotic morphological changes (Figure 2a). That is, the nucleus volume was significantly reduced and apoptotic bodies with blue fluorescence occurred. The fluorescence intensity showed the number of apoptotic bodies increased gradually with the increase of malvidin concentration. In addition, the HSC‐T6 cell volume decreased with malvidin (Figure 2b). Treated with a high concentration of malvidin, cytoplasmic shrinkage, excessive swollen, and remarkable elongation of vacuole‐like structures were observed around the nucleus. Pyknosis and margination of nuclear chromatin on the cell surface and the ER stress injury of cells were also observed in cells with the malvidin more than 75 μg/ml. This indicated that malvidin could induce the HSC‐T6 cell apoptosis. A positive correlation between the cell apoptosis extent/damage degree and the concentration of malvidin existed.

### 3.3 Malvidin induced HSC‐T6 cell apoptosis

**Figure 3:**
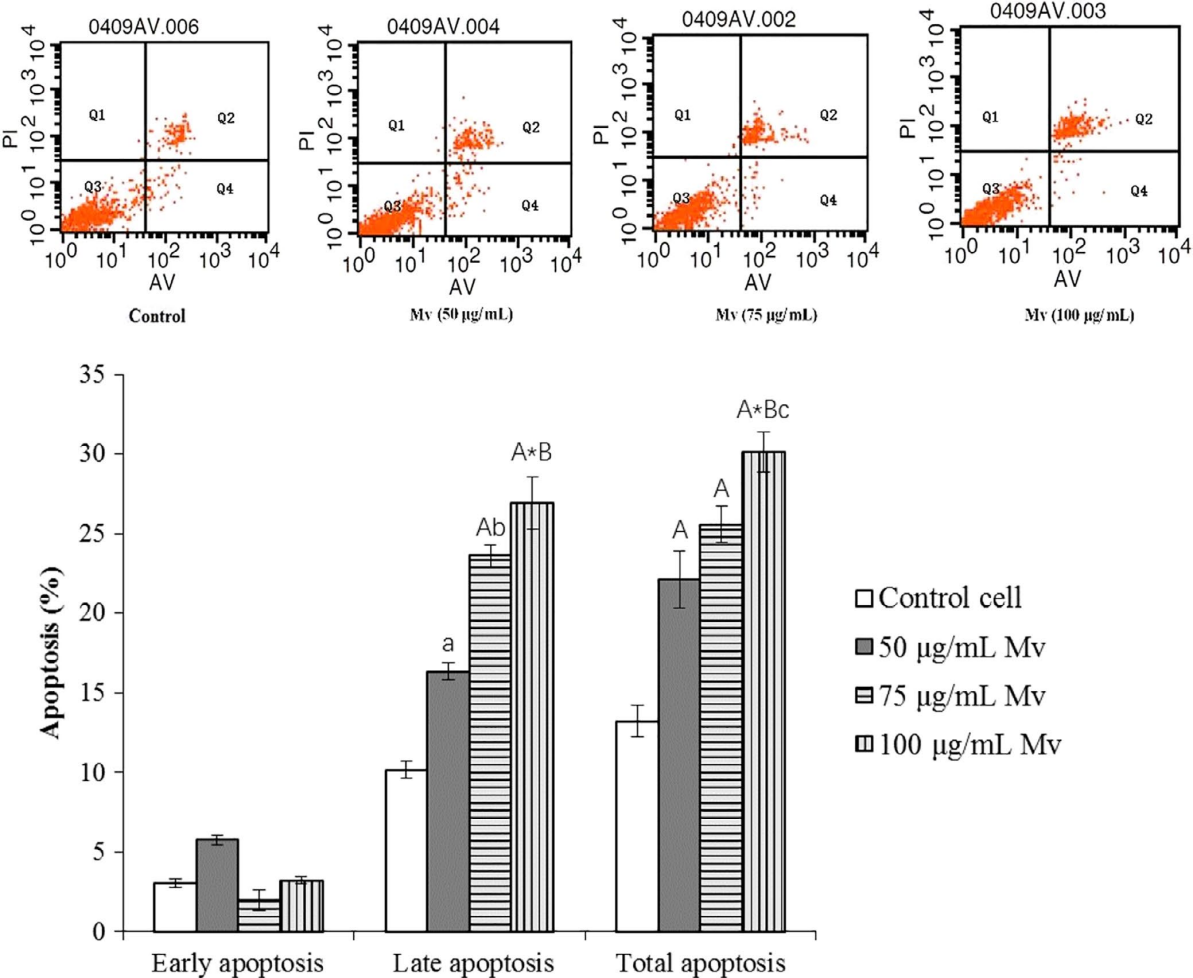
Effects of malvidin on HSC‐T6 apoptosis. HSC‐T6 cells were incubated with various concentrations of malvidin (Mv) for 24 hr. The apoptosis of HSC‐T6 was detected using annexinV‐PI double staining and flow cytometry analysis. A representative set of images from three independent experiments is shown. Results represent viable cells (Q3 quadrant), necrotic cells (Q1 quadrant), early apoptotic cells (Q4 quadrant), and late apoptotic cells (Q2 quadrant). Data are presented as mean ± *SD*. ^a^ *p* < .05, ^A^ *p* < .01, ^A*^ *p* < .001 compared to the control, ^b^ *p* < .05, ^B^ *p* < .01 versus the Mv group (50 μg/ml), ^c^ *p* < .05 versus the Mv group (75 μg/ml)

The HSC‐T6 cell apoptosis was evaluated through the Annexin V/PI assay. After 50, 75, and 100 μg/ml of malvidin treated for 24 hr, the total apoptotic percentages (combined the early and late apoptotic cells) were significantly increased from 13.21 ± 1.01% (the control cells) to 22.11 ± 1.78% (*p* < .01), 25.58 ± 1.12% (*p* < .01), and 30.12 ± 1.26% (*p* < .001), respectively (Figure 3). The control group's late apoptotic rate was 10.17 ± 0.24%. The 50 μg/ml Mv group's was 16.35 ± 0.33% (*p* < .05 vs. the control). The 75 μg/ml Mv group's was 23.61 ± 0.67% (*p* < .01 vs. the control, *p* < .05 vs. the 50 μg/ml Mv group). The 100 μg/ml Mv group's was 26.91 ± 0.22% (*p* < .001 vs. the control, *p* < .01 vs. the 50 μg/ml Mv group). However, there was no significant difference on early apoptotic rates between Mv groups and the control (*p* > .05). Therefore, malvidin mainly induced the HSC‐T6 cell late apoptosis dose‐dependently.

### 3.4 Malvidin triggered HSC‐T6 cell ROS generation

**Figure 4:**
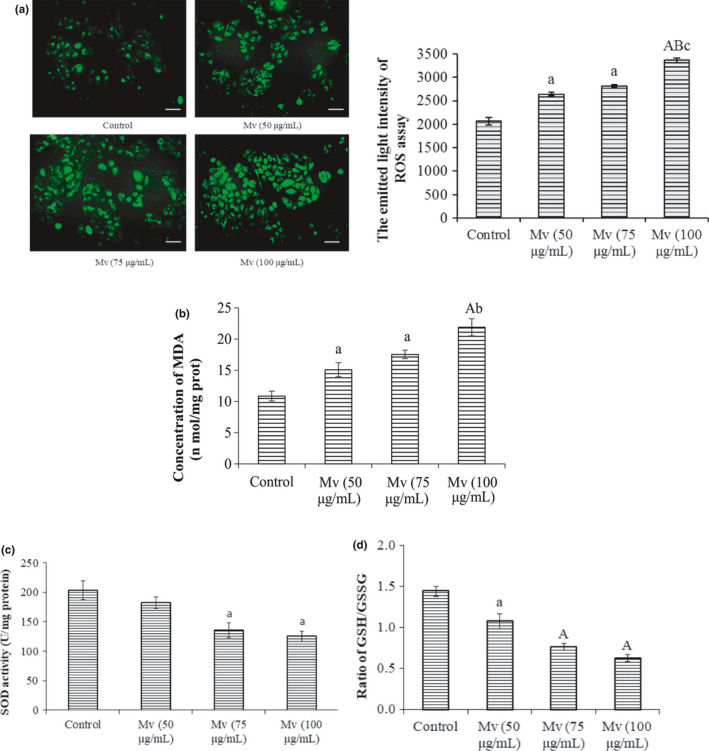
Effects of malvidin on reactive oxygen species (ROS) levels in the HSC‐T6 cells. HSC‐T6 cells were incubated with various concentrations of malvidin for 24 hr. Fluorescence was examined using a fluorescence microscope and levels of ROS were determined by DCFH‐DA assay (a, Scale bar, 50 µm). The MDA levels (b), SOD activity (c), and the ratio of GSH/GSSG (d) were measured by corresponding test kits. Data are presented as mean ± *SD*. ^a^ *p* < .05, ^A^ *p* < .01 compared to the control, ^b^ *p* < .01, ^B^ *p* < .01 versus the Mv group (50 μg/ml), ^c^ *p* < .05 versus the Mv group (75 μg/ml)

Reactive oxygen species, recognized as a well‐known inducer of apoptosis generation, can cause mitochondrial dysfunction and result in membrane depolarization (Ozben, 2007). To clarify if ROS generation involved in malvidin‐induced HSC‐T6 cell apoptosis, a DCFH‐DA probe was applied to examine the ROS production. As shown in Figure 4a, the control cells showed low ROS levels in HSC‐T6 cells with little green fluorescence after DCFH‐DA staining. However, the fluorescent intensity enhanced with the increase of malvidin concentration (*p* < .05), indicating that the ROS levels in malvidin‐induced HSC‐T6 cells were dose‐dependently increased. The Mv groups at concentrations of 50 and 75 μg/ml showed significant difference to the control with 1.28 and 1.36 folds of ROS levels (*p* < .05), while the Mv group at concentrations of 100 μg/ml showed extremely significant differences to the control with 1.63 folds of ROS level (*p* < .01). This indicated that Mv triggered ROS generation and ROS played roles in Mv‐induced apoptosis.

In addition, malondialdehyde, an oxidative damaged product of lipid peroxidation, which acts as a crucial indicator of the redox potential in cells, was also detected after incubation with malvidin for 24 hr. Results showed that the MDA content was dose‐dependently upregulated in HSC‐T6 cells after malvidin treatment (Figure 4b). The MDA levels in cells treated with 50, 75, and 100 μg/ml Mv significantly increased from 10.86 ± 0.80 (the control) to 15.09 ± 1.12, 17.55 ± 0.67, and 21.88 ± 1.36 nmol/mg protein (*p* < .05). Also, a high level of malvidin (100 μg/ml) induced a significantly higher MDA level (*p* < .05) than a low level (50 μg/ml).

The overproduction of ROS triggers serious damages in various cells, related to increased MDA levels and decreased SOD and GSH contents. Therefore, the SOD activity and the GSH/GSSG ratio were also measured. Our results demonstrated that malvidin‐induced overproduction of ROS led to a dose‐dependent decrease of SOD activity and GSH/GSSG ratio (Figure 4c,d). Compared to the control, the SOD levels in cells treated with 75 and 100 μg/ml Mv significantly decreased from 203.50 ± 15.72 to 135.55 ± 12.43 and 125.33 ± 6.67 U/mg protein (*p* < .05), while the GSH/GSSG ratio decreased from 1.45 ± 0.06 to 0.77 ± 0.043 and 0.63 ± 0.04 (*p* < .05). These results provided evidence for the malvidin‐induced ROS overproduction.

### 3.5 Malvidin induced apoptosis through an endoplasmic reticulum stress pathway and a mitochondrial pathway

Endoplasmic reticulum stress (ERS) is important for the interplay of apoptosis in many cells (Cheng et al., 2018). Excessive ERS can induce cell apoptosis by activating critical mediators, caspase‐12 and C/EBP homologous protein (CHOP; Görlach, Klappa, & Kietzmann, 2006). The endoplasmic reticulum molecular chaperone GRP78 is also connected to ERS‐mediated apoptotic pathway. Considering the changes on the structure of endoplasmic reticulum in cells by TEM analysis, caspase‐12, CHOP, and GRP78 protein expression were detected by Western blot to investigate if ERS involved in the apoptotic process of Mv‐induced HSC‐T6 cells. Moreover, the mitochondria‐mediated intrinsic apoptotic signaling proteins, the Bcl‐2, Bax, and caspase‐3 were also detected.

**Figure 5:**
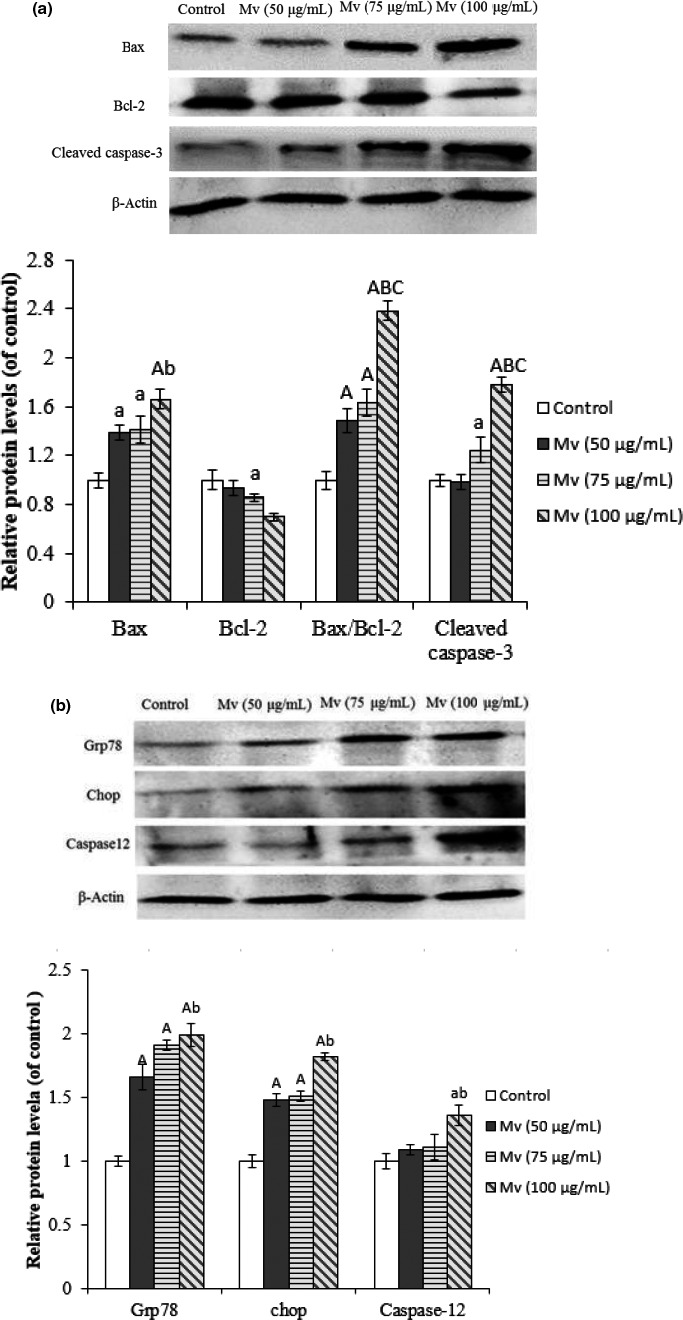
Western blot analysis of apoptosis‐related protein expression, including Bax, Bcl‐2, Bax/Bcl‐2, and cleaved caspase‐3 (a) and GRP78, CHOP, and caspase‐12 (b). Data are presented as mean ± *SD*. ^a^ *p* < .05, ^A^ *p* < .01 compared to control, ^b^ *p* < .05, ^B^ *p* < .01 versus the Mv group (50 μg/ml), ^C^ *p* < .01 versus the Mv group (75 μg/ml). Representative Western blots are shown

Malvidin downregulated the anti‐apoptotic Bcl‐2 level, while upregulated pro‐apoptotic Bax and caspase‐3 levels in HSC‐T6 cells dose‐dependently (Figure 5a). HSC‐T6 cells with 100 μg/ml of malvidin possessed the largest changes, whose Bcl‐2, Bax, and cleaved caspase‐3 protein expression levels were 0.69 ± 0.03, 1.66 ± 0.08, and 1.78 ± 0.06 folds of the control (all *p* < .01), respectively. Bax/Bcl‐2 ratio may be more useful in determining apoptosis than each promoter. The values of the control, 50, 75, and 100 μg/ml Mv groups on the Bax/Bcl‐2 ratio were 1, 1.49, 1.64, and 2.38, respectively. As shown in Figure 5b, malvidin at all the concentrations significantly increased the GRP78 and CHOP expression levels (all *p* < .01), which were about 1.5–2.0 folds of the control. Only a high concentration of malvidin (100 μg/ml) could significantly increase caspase‐12 protein expression to 1.37 ± 0.08 folds of the control (*p* < .05). In addition, the 50 μg/ml Mv group had significantly different protein levels compared with the 100 μg/ml Mv group (*p* < .05). All these indicated that a high concentration of malvidin induced HSC‐T6 cell apoptosis by an ERS pathway and a mitochondrial pathway, which might be the main reason for the decrease of HSC‐T6 cells viability.

## 4 DISCUSSION

Liver fibrosis, as consequence of chronic damage in the liver, causes the extracellular matrix protein accumulation (Lee & Friedman, 2011). According to recent findings, the elimination of activated HSCs and the induction of HSC apoptosis might be a crucial mechanism to terminate and potentially reverse hepatic fibrosis (Zhang et al., 2013). Anthocyanins from blueberries, one of important antioxidant resources, possess powerful health‐promoting benefits. Many studies have proved the negative relationship between anthocyanin intake and disease risk, including cancers, chronic diseases, neurodegenerative conditions, as well as hepatic fibrosis (Braga, Murador, de Souza Mesquita, & de Rosso, 2018; Cassidy et al., 2013; Teng et al., 2017). The protective effect of anthocyanins to human body was related to the quantity of anthocyanin intake. Guo et al. (2020) reported that anthocyanin supplementation at a dose greater than 80 mg/day is an effective agent against metabolic risk factors including oxidative and inflammatory biomarkers in healthy young adults. Barfoot et al. (2019) reported that children aged 7–10‐year‐old could further improve their learning and cognitive function when they intake an anthocyanin‐rich wild blueberry drink (253 mg anthocyanins). However, the dosage of anthocyanins used in the human intervention trials varies widely (100–640 mg/day) for unhealthy individuals (Qin et al., 2009; Yang et al., 2017; Zhang, Chen, Li, Ling, & Guo, 2015). High doses (≥320 mg/day) of dietary anthocyanin supplements were generally required to alleviate oxidative stress and inflammation and improve the metabolic profile in patients with chronic diseases (Li, Zhang, Liu, Sun, & Xia, 2015). The bioavailability of anthocyanin is usually very low (<1% of the initial doses; Santhakumar, Battino, & Alvarez‐Suarez, 2018). Numerous studies have shown that low concentration of anthocyanins in plasma and urine has been detected (Hidalgo et al., 2012). Despite its bioavailability controversies, anthocyanin intake exhibits promising results as effective complementary therapeutic alternatives in reducing health threat of high‐risk populations. Previous reports have demonstrated that anthocyanins could play hepatoprotective and anti‐fibrotic effects by regulating oxidative stress, suppressing HSC proliferation, inhibiting multiple signaling pathways, including the TGF‐β1, TNF‐α, PDGFR‐β, Akt, and ERK1/2 pathways (Choi et al., 2011; Connolly et al., 2009). Nevertheless, there have been no reports on the apoptosis induced by anthocyanins and their possible mechanism in HSCs. Here, the abundant anthocyanin in blueberries, malvidin's effects on activated HSC‐T6 cell apoptosis and its mechanism were investigated.

Based on the MTT assay, LDH activity measure, and morphological change observation, malvidin was found to efficiently attenuate HSC‐T6 cell proliferation, enhance cell necrosis, and induce the apoptotic occurrence of activated HSC‐T6 cells dose‐dependently. Apoptosis might be the main reason for the decrease of cell proliferation and the increase of cell necrosis. Number of apoptotic bodies and degree of cytoplasmic shrinkage were observed in malvidin‐treated HSC‐T6 cells. A high concentration of malvidin exhibited more pronounced apoptotic effects to HSC‐T6 cells than a low concentration. Similarly, Choi et al. (2011) previously reported that the purple sweet potato anthocyanins concentration‐dependently suppressed the hepatic stellate cell proliferation.

Reactive oxygen species have been recognized as a potential apoptosis modulator. Powerful evidences prove that cancer cells generally overproduce ROS resulting in oxidative stress (Jeelani et al., 2017). Among many ROS production resources, mitochondrion is one important and major source. The increase of mitochondrion ROS levels can induce the mitochondrial membrane potential collapse, release LDH, and increase lipid peroxidation product MDA (Zhao & Yu, 2018), which will trigger a series of mitochondria‐associated events, such as activating caspase‐9 (the initiator), subsequently caspase‐3, caspase‐6, and caspase‐7 (the effector), eventually leading to apoptosis (Noh et al., 2015; Zou et al., 2018). However, the roles of ROS on mitochondrion apoptosis pathway are still unknown in Mv‐induced HSC‐T6 cells. In this study, ROS level, MDA concentration, and LDH release were found to increase at dose‐dependent manner after Mv treatment, which was consistent with the results of apoptosis. Therefore, the oxidative stress due to ROS and MDA overproduction might be one reason for HSCs apoptosis.

Additionally, SOD is a key antioxidant enzyme, which catalyzes the disproportionation of superoxide (O^2−^) radicals into H_2_O_2_ and O_2_. Furthermore, reduced glutathione (GSH) is the most abundant intracellular and multifunctional antioxidant present in all the living organisms. In normal conditions, oxidized glutathione disulfide (GSSG) is maintained at the concentration lower than 5% of the total glutathione content in the cell. During oxidative stress, intracellular GSH is depleted with a concomitant increase of its oxidized form. The GSH/GSSG ratio has been used as an important biomarker of the redox balance in the cell and consequently of cellular oxidative stress in vitro and in vivo. Jia et al. (2018) reported that ROS accumulated significantly in the activated HSCs under curcumol treatment, and ROS overexpression might be the key mechanism of curcumol‐induced necroptosis to inhibit HSC activation. Our results demonstrated that malvidin‐induced overproduction of ROS led to a decrease of SOD activity and GSH/GSSG ratio. These findings collectively indicated that ROS overexpression triggered the imbalance of oxidize and anti‐oxidize system in the activated HSCs, which might be the key mechanism of malvidin‐induced HSCs cell apoptosis.

Interestingly, malvidin, as a potential antioxidant in vitro, could protect some normal cells (e.g., human retinal pigment epithelial cells) from oxidative stress by decreasing ROS and MDA productions and inhibiting cell apoptosis in a lower concentration (5 μg/ml; Huang et al., 2018). However, high concentrations of malvidin (50–100 μg/ml) could increase ROS and MDA levels and induce apoptosis in activated hepatic stellate cells here. Sun et al. (2018) found that blueberry anthocyanins significantly inhibited the hepatic stellate cell proliferation at high concentrations (100–200 mg/ml). Similarly, previous studies reported malvidin's cytotoxicity to various tumor cells in vitro or in vivo in a high concentration (Mazewski et al., 2018; Yoshino et al., 2018). Feng et al. (2007) found that a high dose of cyanidin‐3‐rutinoside, another anthocyanin, also selectively induced leukemic cell apoptosis by inducing oxidative stress. Of course, it is necessary to estimate toxicity of different concentration anthocyanin to normal hepatocytes when studying the anti‐hepatic fibrosis effect of malvidin on HSCs in vitro. Our preliminary studies revealed that high concentrations of malvidin selectively inhibited HSCs’ cell proliferation, but only had minor inhibitory effect on rat hepatocytes (unpublished data). These results indicated that anthocyanins are widely available and have the advantages to inhibit abnormal cells selectively, such as cancer cells, as well as HSCs. Anthocyanins have different effects on the physiologic parameters depending on the change of concentration. Sometime they are beneficial for the cells, while they are detrimental to the cells on the other concentration (Chen et al., 2014). Thus, concentration windows should be considered when anthocyanins are used to treat liver fibrosis and cancer as pharmaceutical ingredients or nutraceuticals.

In general, the mitochondrial signaling pathway is the intrinsic pathway, which is involved in some apoptosis induced by natural products (Pan et al., 2017). Caspase cascade activation is a crucial affair in the mitochondrial apoptotic pathways. Caspase‐3 is a key indicator in mitochondrial pathway‐induced cell apoptosis, which play critical roles in initiating the caspase cascade reaction (Joza et al., 2008). Additionally, the proteins in Bcl‐2 family are important to modulate the mitochondria‐related apoptotic pathway (Chipuk et al., 2004). Bcl‐2 inhibits Cyt C release, while Bax promotes the Cyt C release in the mitochondria (Elmore, 2007). The Bax/Bcl‐2 ratio is used to evaluate the death or survival, which reflects maintenance of mitochondrial membrane stability after an apoptotic stimulus (Ghribi, DeWitt, Forbes, Herman, & Savory, 2001). An elevated Bax/Bcl‐2 ratio indicates the cell apoptosis outbreak. In the present study, high concentrations of malvidin decreased the Bcl‐2 level, while increased the Bax level and the Bax/Bcl‐2 ratio. Moreover, malvidin also enhanced the expression of cleaved caspase‐3, confirming the increase of cell apoptosis. The data above demonstrated that malvidin induced HSCs apoptosis through mitochondrial apoptosis pathway.

The endoplasmic reticulum (ER), one organelle important for signal transduction and protein metabolism in eukaryotic cells, contributes to the protein synthesis and transshipment in cells and serves as a pool to store calcium (Fribley, Zhang, & Kaufman, 2009). Recent researches found the ability of ER under pressure to initiate the intrinsic pathway (Zielinski, Eigl, & Chi, 2013). However, the correlation between apoptosis and ERS has still been unknown. Caspase‐12, CHOP, and GRP78 are ERS relative proteins. Caspase‐12 is an ERS‐induced apoptotic marker (Nakagawa & Yuan, 2000). CHOP is the most important pro‐apoptotic protein to initiate ERS‐induced apoptosis (Kim, Xu, & Reed, 2008). GRP78 is dissociated with the accumulation of unfolded proteins under ERS conditions (Nakagawa & Yuan, 2000). Prolonged ERS is able to activate the caspase‐12 and CHOP. In the present study, the caspase‐12, CHOP, and GRP78 levels were all upregulated after malvidin treatment. These findings proved malvidin induced HSC apoptosis through the ERS‐mediated pathway. That might explain that the overwhelmed ERS could greatly increase cell injury and ultimately induce cell death.

## 5 CONCLUSIONS

Malvidin could inhibit cell proliferation and induce cell apoptosis dose‐dependently in HSC‐T6 cells. A high concentration of malvidin significantly evaluated ROS level and MDA concentration. At the same time, malvidin triggered mitochondrial dysfunction by activating caspase‐3, downregulating Bcl‐2, and upregulating Bax. Moreover, malvidin activated endoplasmic reticulum‐related signals in HSC‐T6 cells, for example, caspase‐12, CHOP, and GRP78. Overall, the higher concentration of malvidin had more pronounced effects. The results indicated that malvidin could induce HSC apoptosis via the mitochondrial‐dependent pathway and ERS pathway. These findings would provide a clue for blueberry anthocyanins, especially malvidin, served as a potential nutraceutical to benefit for the liver health. In the future, studies in vitro/in vivo on the functions and mechanisms of blueberry anthocyanins against hepatic fibrosis would be furtherly conducted.

## CONFLICT OF INTEREST

There is no conflict of interests regarding the publication of this paper.
